# Effect of Biotic Elicitors on the Growth, Antioxidant Activity and Metabolites Accumulation in In Vitro Propagated Shoots of *Pueraria tuberosa*

**DOI:** 10.3390/plants12061300

**Published:** 2023-03-14

**Authors:** Bhanupriya Kanthaliya, Abhishek Joshi, Jaya Arora, Mashael Daghash Alqahtani, Elsayed Fathi Abd_Allah

**Affiliations:** 1 Laboratory of Biomolecular Technology, Department of Botany, Mohanlal Sukhadia University, Udaipur 313001, Rajasthan, India; 2 Department of Biology, College of Science, Princess Nourah bint Abdulrahman University, P.O. Box 84428, Riyadh 11671, Saudi Arabia; 3 Plant Production Department, College of Food and Agricultural Sciences, King Saud University, P.O. Box 2460, Riyadh 11451, Saudi Arabia

**Keywords:** elicitor, secondary metabolite, isoflavonoids, antioxidant, biomass

## Abstract

*Pueraria tuberosa* contains a wide range of bioactive compounds, including polyphenols, alkaloids, and phytosterols, which make it valuable to the pharmaceutical and food industries. Elicitor compounds trigger the defense mechanisms in plants and are widely used to increase the yield of bioactive molecules in in vitro cultures. The present study was conducted to evaluate the effects of different concentrations of biotic elicitors such as yeast extract (YE), pectin (PEC), and alginate (ALG) on growth, antioxidant activity, and metabolite accumulation in in vitro propagated shoots of *P. tuberosa*. The elicitors applied to shoot cultures of *P. tuberosa* significantly increased biomass (shoot number, fresh weight, and dry weight), and metabolites such as protein, carbohydrates, chlorophyll, total phenol (TP), and total flavonoid (TF) contents, as well as antioxidant activity compared to untreated control. Biomass, TP, and TF contents, as well as antioxidant activity, were most significant in cultures treated with 100 mg/L PEC. In contrast, chlorophyll, protein, and carbohydrate increased most in cultures treated with 200 mg/L ALG. Application of 100 mg/L of PEC led to the accumulation of high amounts of isoflavonoids including puerarin (220.69 μg/g), daidzin (2935.55 μg/g), genistin (5612 μg/g), daidzein (479.81 μg/g), and biochanin-A (111.511 μg/g) as analyzed by high-performance liquid chromatography (HPLC). Total isoflavonoids content of 100 mg/L PEC treated shoots was obtained as 9359.56 μg/g, 1.68-fold higher than in vitro propagated shoots without elicitors (5573.13 μg/g) and 2.77-fold higher than shoots of the mother plant (3380.17 μg/g). The elicitor concentrations were optimized as 200 mg/L YE, 100 mg/L PEC, and 200 mg/L ALG. Overall, this study showed that the application of different biotic elicitors resulted in better growth, antioxidant activity, and accumulation of metabolites in *P. tuberosa*, which could lead to obtaining phytopharmaceutical advantages in the future.

## 1. Introduction

*Pueraria tuberosa* (Roxb. Ex Willd.) DC., known as “Kudzu”, belongs to the family Fabaceae and is native to South-East Asia. Kudzu is considered the most popular medicinal plant in traditional Chinese medicine and Ayurveda [1]. It has also received attention in modern pharmacopoeia due to the presence of numerous bioactive compounds, such as polyphenols, alkaloids, and phytosterols. Its tubers contain 49 different isoflavonoids. Of these, the five major isoflavonoids are puerarin, daidzin, genistin, daidzein, and biochanin-A [2]. Dietary intake of isoflavonoids has a major impact on human health, and they are capable of reducing osteoporosis risk, attenuating menopause symptoms, possessing anticancer, antidiabetic, anti-inflammatory, neuroprotective, wound healing, hypolipidemic, and nootropic properties [3,4].

In plants, the production of secondary metabolites is influenced by many factors, including genotype or variety, plant parts, growth periods, propagation methods, and environmental conditions [5]. In vitro plant tissue or cell culture techniques can be used to produce genetically uniform plantlets with homogenous metabolite contents within a short period. In response to the industrial demand for plant metabolites, this is the most effective way to increase production without compromising sustainability [6]. Several strategies can be used to increase secondary metabolite production in plant tissues and cell culture [7,8]. The employment of biotic or abiotic elicitors has emerged as a vital biotechnological approach to drive mass production and obtain the desired metabolite concentrations. Elicitors are a group of compounds that trigger defense mechanisms in plants and result in the formation of secondary metabolites [9,10]. These compounds also act as antioxidants and scavenge free radicals and reactive oxygen species [11,12]. 

The most common biotic elicitors are pectin (PEC), alginate (ALG), and yeast extract (YE). These elicitors are commonly called polysaccharide elicitors and are extracted, isolated, and purified from the cell walls of living organisms, either plants or microorganisms [13,14]. Different compounds present in these elicitors have been implicated in plant defense responses, including the activation of signaling pathways, ethylene production, and inhibition of auxin-induced responses [15,16]. It has been used successfully in both organized and unorganized cultures of plants to increase the production of miscellaneous bioactive molecules [17,18].

Many empirical studies have focused on developing effective strategies to increase the concentration of bioactive molecules in *Pueraria* species [19,20,21]. Some researchers have reported improved accumulation of isoflavonoids by prioritizing macro- and micronutrients and precursors in the culture medium for *P. tuberosa* [22,23,24]. Goyal and Ramawat [25] reported a marked increase in isoflavonoids production in cell suspension culture of *P. tuberosa* by elicitation treatment with YE, methyl jasmonate, and salicylic acid. In this study, 150 mg/L YE was found to be optimal for isoflavonoid production, yielding 10 mg/L isoflavonoids. Another study reported improved isoflavonoid accumulation using *Cuscuta reflexa* as an elicitor for *P. tuberosa* cell cultures [26]. A maximum yield of 91 mg of isoflavonoids was recorded at 1 g/L, which was approximately 19% higher than that in the control cultures. Evidence also suggests that elicitation adversely affects numerous physiological and biochemical processes, which depend on elicitor concentration, chemical nature, treatment duration, and plant species [27,28]. It is, however, necessary to determine the optimal elicitor concentration in order to prevent oxidative stress, which can result in cell death due to excessive elicitor levels. Furthermore, a thorough understanding of the biosynthesis route of bioactive molecules in in vitro propagated plantlets without adversely affecting their growth will be useful for screening and developing plant varieties with higher amounts of secondary metabolites. Therefore, the present study aimed to determine the effects of biotic elicitors (YE, PEC, and ALG) on growth, chlorophyll, protein, carbohydrates, total phenols (TP), total flavonoids (TF), and antioxidant activity in in vitro propagated shoots of *P. tuberosa,* and to obtain information about the optimal treatments for both plant growth and bioactive molecule production.

## 2. Results

### 2.1. Effect of Elicitor Treatments on Growth Parameters

**Figure 1:**
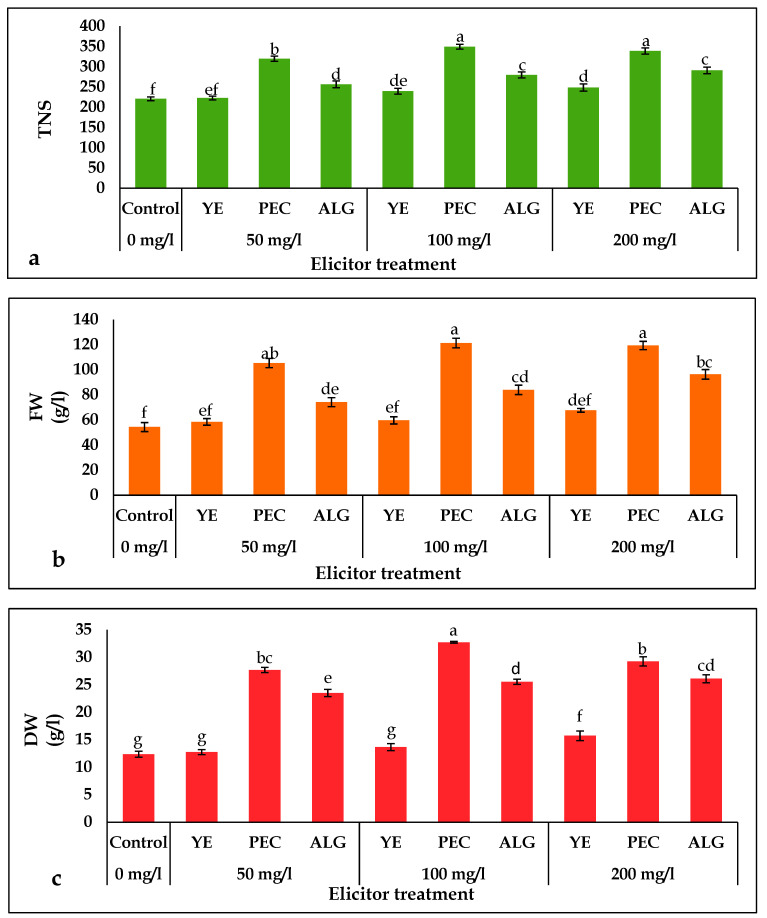
Effect of elicitor treatments on morphological attributes in in vitro shoots of *P. tuberosa*: (**a**) TNS, (**b**) FW (g/L), (**c**) DW (g/L). Values are mean ± SD, means in a column followed by different letters are significantly different from each other at *p* < 0.05. Mean values followed by the same letter(s) in a column are not significantly different (*p* < 0.05) based on Duncan multiple range test (DMRT).

*P. tuberosa* shoot cultures treated with different concentrations (50, 100, 200 mg/L) of YE, PEC, and ALG showed a significant increase in total number of shoots (TNS), fresh weight (FW), and dry weight (DW) at all concentrations of elicitor (Figure 1). The PEC treated plantlets showed the highest number of shoots, 349.48 shoots per liter of MS medium at 100 mg/L of PEC concentration, which was 1.5-fold higher than that of the control. The most pronounced increases in FW (2.2-fold) and DW (2.4-fold) were observed in cultures supplemented with 100 mg/L PEC compared to the control. The lowest increase in the production of TNS, FW, and DW was detected in the 50 mg/L YE treated plantlets as compared with other treatments. PEC was optimal for biomass (TNS, FW, and DW) at 100 mg/L, whereas YE and ALG were optimal at 200 mg/L.

### 2.2. Effect of Elicitor Treatments on Chlorophyll, Protein, and Carbohydrate Content

The accumulation of chlorophyll (Chl a, Chl b, and Total Chl), soluble protein, and carbohydrate content in cultures supplemented with 50, 100, and 200 mg/L of YE, PEC, and ALG was higher than in the control cultures (Table 1). The most pronounced increase in Chl a was observed in cultures treated with 100 mg/L PEC, which was approximately 1.6-fold greater than that in the untreated control. The ALG treated plantlets showed the highest accumulation of Chl b and Total Chl, 18.87 and 48.5 mg/g at 200 mg/L ALG concentration, which were 1.63-fold and 1.57-fold higher than control, respectively. The maximum increase in protein and carbohydrate content appeared in cultures supplemented with 200 mg/L ALG, which were approximately 2.3-fold and 1.75-fold greater than in control cultures, respectively. 

### 2.3. Effect of Elicitor Treatments on Total Phenolics (TP) and Total Flavonoids (TF) Contents 

**Figure 2:**
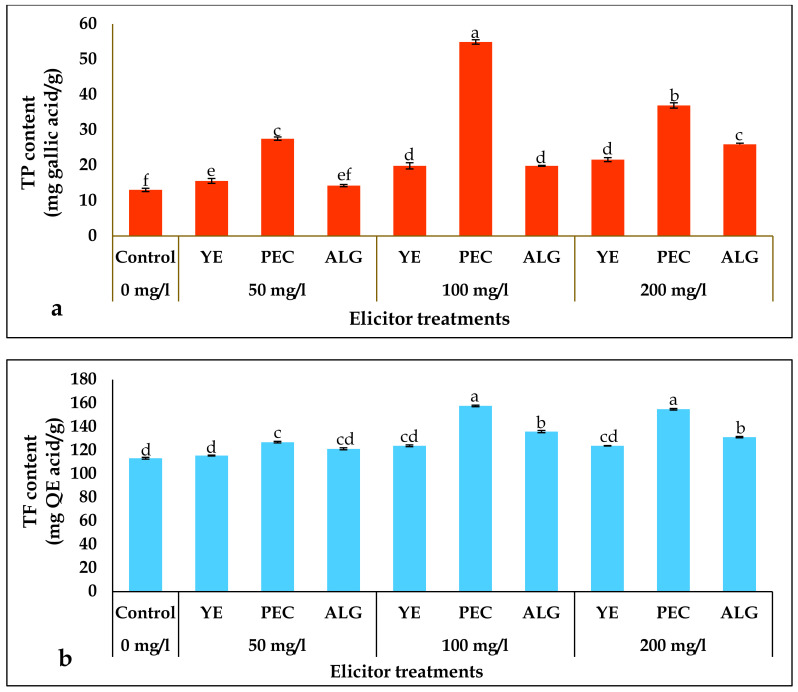
Effect of elicitor treatments on TP and TF contents in *P. tuberosa* shoots: (**a**) TP content (mg gallic acid/g), (**b**) TF content (mg QE acid/g). Note: Values are mean ± SD, means in a column followed by different letters are significantly different from each other at *p* < 0.05. Mean values followed by the same letter(s) in a column are not significantly different (*p* < 0.05) based on DMRT.

The TP and TF contents of the cultures treated with 50, 100, and 200 mg/L of YE, PEC, and ALG were greater than those of the control (Figure 2). The highest TP accumulation was observed in cultures supplemented with 100 mg/L PEC, which was approximately 4.2-fold higher than that in the control cultures. The TF content of cultures significantly increased after treatment with all elicitor concentrations. The highest TF content was observed in cultures treated with 100 mg/L PEC, which was approximately 1.4-fold higher than that in the untreated control. The lowest TP and TF contents were detected in cultures treated with 50 mg/L YE compared to other concentrations of YE, PEC, and ALG. The optimal concentration of PEC for TP and TF content was 100 mg/L, whereas for YE and ALG it was 200 mg/L.

### 2.4. Effect of Elicitor Treatments on Antioxidant Activity

**Figure 3:**
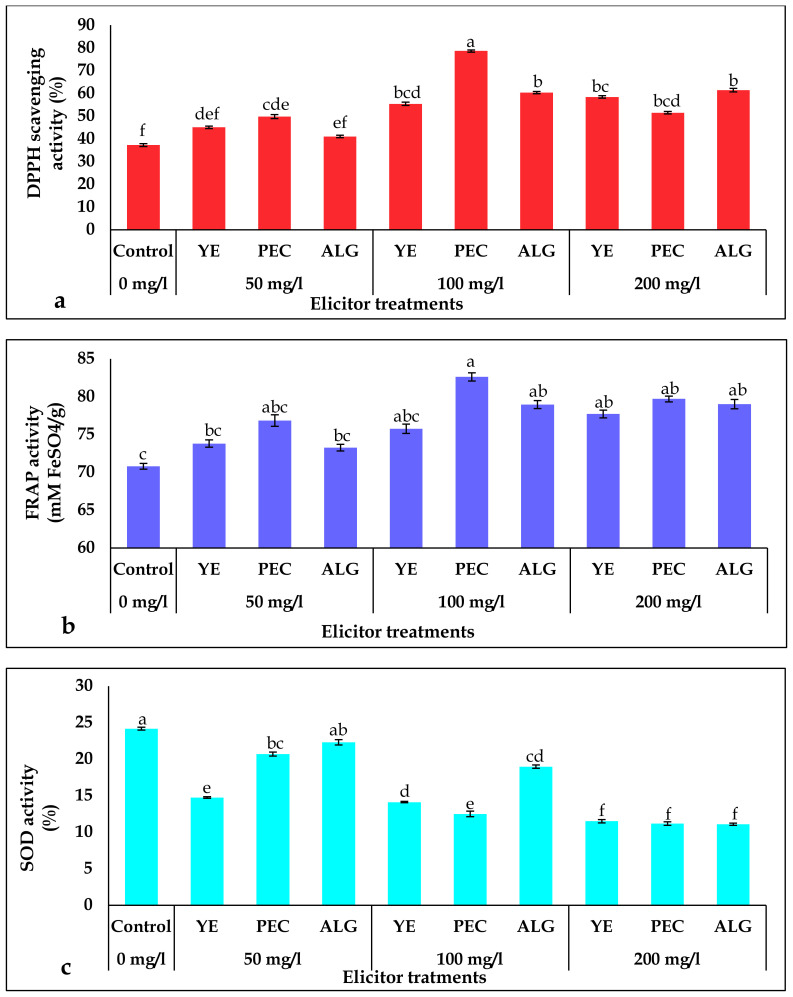
Effect of elicitor treatments on antioxidant activity in *P. tuberosa s*hoots: (**a**) DPPH activity (%), (**b**) FRAP activity (mM FeSO_4_/g), (**c**) SOD activity (%). Note: Values are mean ± SD, means in a column followed by different letters are significantly different from each other at *p* < 0.05. Mean values followed by the same letter(s) in a column are not significantly different (*p* < 0.05) based on DMRT.

The three assays, DPPH (1,1-Diphenyl-2-picrylhydrazyl), FRAP (ferric reducing antioxidant potential), and SOD (superoxide dismutase), were used to estimate the antioxidant activity in *P. tuberosa* shoot culture extract after elicitation with 50, 100, and 200 mg/L of YE, PEC, and ALG (Figure 3). The DPPH and ferric radical scavenging potential of the shoot cultures significantly increased after treatment with all elicitor concentrations. The most pronounced increase in DPPH and ferric radical scavenging activity was observed in cultures treated with 100 mg/L PEC, which were 2.1-fold and 1.1-fold higher than the control cultures, respectively. The lowest increase in DPPH and FRAP activity was found in the 50 mg/L ALG treated cultures compared to other applied dosages of elicitors. In contrast, SOD activity in the culture decreased with increasing elicitor concentration. The highest SOD activity was 22.3% at a 50 mg/L concentration of ALG. The lowest SOD activity was detected at the highest concentration in all treatments (200 mg/L).

### 2.5. HPLC Analysis

**Figure 4:**
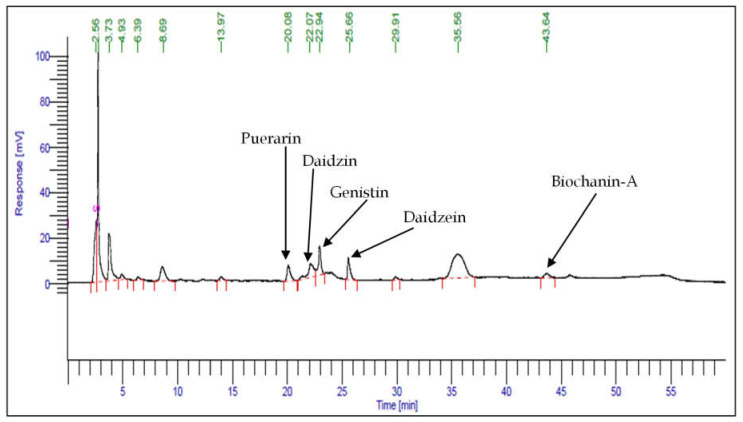
HPLC chromatogram of PEC elicited (100 mg/L) shoots of *P. tuberosa*.

PEC at 100 mg/L concentration resulted in the highest TP and TF contents, as well as antioxidant activity among all elicitor treatments. Based on these preliminary results, 100 mg/L PEC treated shoot cultures were harvested and HPLC analysis was performed. Major isoflavonoids puerarin (220.69 μg/g), daidzin (2935.55 μg/g), genistin (5612.00 μg/g), daidzein (479.81 μg/g), and Biochanin-A (111.511 μg/g) were obtained (Figure 4). The total isoflavonoid content obtained from 100 mg/L PEC treated shoots was 9359.56 μg/g, which was 1.68-fold higher than that of in vitro propagated shoots without elicitors (5573.13 μg/g) and 2.77-fold higher than that of the shoots of the mother plant (3380.17 μg/g).

### 2.6. Principal Component Analysis

**Figure 5:**
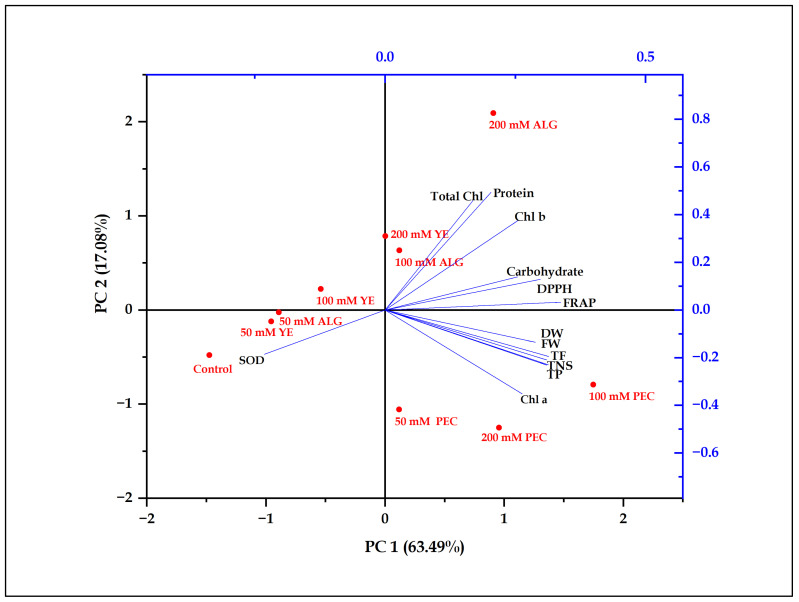
Principal component analysis of physio-biochemical indices of *P. tuberosa* shoots for different elicitor treatments.

The effects of different elicitor treatments on physio-biochemical indices of *P. tuberosa* shoots were evaluated by principal component analysis (PCA). The components PC1 and PC2 explained 63.49% and 17.08% of the overall variance, respectively (Figure 5). The significant effects of the elicitor treatments on the studied attributes were clearly distributed along the PC1 axis in the order 100 mM PEC > 200 mM ALG > 200 mM PEC > 50 mM PEC > 100 mM ALG > 200 mM YE > 100 mM YE > 50 mM ALG > 50 mM YE > Control. PEC treatments scored significantly higher on PC1 and were clustered on the right side, whereas lower scoring treatments were clustered on the left side. PCA revealed better physio-biochemical indices in PEC treated shoots among all elicitor treatments tested. A loading matrix was used to determine the degree of correlation between physio-biochemical traits and a specific principal component (Table 2). PC1 demonstrated significant positive relationships and high loading conditions for all physio-biochemical parameters. PC2 exhibited significant positive relationships with physio-biochemical indices, but negative relationship were observed in terms of TNS, FW, DW, Chl a, TP, and TF contents. 

## 3. Discussion

There is sufficient evidence that the culture medium composition greatly influences the micropropagation of *Pueraria* species and determines the growth, development, and metabolite composition of plant tissues [29]. The use of biotic elicitors has been shown to facilitate cell growth and increase the accumulation of bioactive molecules within cells and tissues [30,31,32]. Elicitor concentration is a crucial aspect of productive elicitation, and plant species differ in their optimal concentration [33]. In this study, different concentrations of YE, PEC, and ALG were evaluated for their effects on the growth of *P. tuberosa* shoots, as well as their metabolite compositions, such as protein, carbohydrate, chlorophyll, phenolic, and flavonoid contents, and antioxidant activity.

The cell or tissue biomass is one of the most critical aspects of in vitro propagation. Different elicitors have a substantial impact on cell or tissue growth and differentiation [34]. In order to prevent oxidative stress and cell death caused by excessive levels of elicitors, it is necessary to determine the optimal elicitor concentration [35]. In this study, all concentrations of elicitors significantly increased TNS, FW, and DW compared with the control (Figure 1). The applied elicitor treatments in the current study improved growth parameters because natural polysaccharides maintained turgor pressure within the cells and accelerated cell proliferation [36]. Cultures treated with 100 mg/L PEC showed the greatest increases in TNS, FW, and DW. Polysaccharide PEC is a vital component of the cell wall and stimulates many cell wall properties such as porosity, surface charge, ion balance, and lignin biosynthesis [37,38]. This means that the addition of PEC strengthens the cell wall, making them less permeable to pathogens. Therefore, PEC treatment resulted in better shoot growth and increased shoot weight. In a recent study by Haas et al. [39], pectin nanofilaments were found to regulate the shape of plant epidermal cells and to initiate morphogenesis. Previously, a positive effect of YE, PEC, and ALG on in vitro plant growth was demonstrated in many species [40,41].

In the present study, in vitro propagated shoots grown in the presence of elicitors tended to have much greater chlorophyll, protein, and carbohydrate contents than shoots propagated in elicitor-free control medium (Table 1). PEC at 100 mg/L showed the highest accumulation of Chl a, whereas ALG at 200 mg/L showed the highest accumulation of Chl b and Total Chl. The enhanced chlorophyll content in elicitor treated shoots might be due to a constructive effect of the elicitor compounds on the efficiency of the photosynthetic apparatus. There is evidence that naturally occurring polysaccharides boost net photosynthetic rates, stomatal conductance, internal CO_2_ concentrations, and carbonic anhydrase activity in plants [42,43]. The application of 200 mg/L ALG resulted in the highest accumulation of proteins and carbohydrates in the current study. Alginate oligosaccharides promote auxin biosynthesis and calcium signaling; therefore, as an elicitor, their role in increasing metabolites, such as proteins and carbohydrates, can be correlated with enhanced plant growth [44,45].

Phenolic and flavonoid compounds are generally produced through the phenylpropanoid pathway. These compounds do not directly affect plant growth and development, but play defensive roles under biotic or abiotic stress [46,47].

In the current study, cultures treated with elicitors had higher TP and TF contents than untreated control cultures (Figure 2). Cultures supplemented with 100 mg/L PEC accumulated the highest TP and TF contents, approximately 4.2-fold and 1.4-fold higher, respectively, than the untreated controls. This indicates that PEC treatment stimulates phenol and flavonoid production in *P. tuberosa* shoots, which may be due to the overexpression of enzymes associated with polyphenol metabolism. However, it remains unclear how and which biosynthetic enzymes are responsible for phenol and flavonoid overproduction. Previous studies have identified polysaccharides as promising candidates for increasing the levels of phenolic compounds and flavonoids by stimulating the regulatory enzymes of phenylpropanoid metabolism, particularly phenyl ammonia lyases and chalcone isomerase [48,49]. These results are consistent with those of previous studies that showed that high PEC and YE concentrations significantly increased TP and TF levels in cell cultures of Hassawi rice [50] and *Phoenix dactylifera* [51]. 

The cellular machinery of plants produces a variety of enzymatic and non-enzymatic antioxidants to scavenge harmful reactive oxygen species (ROS), which damage DNA, disrupt metabolism, and affect plant growth [52]. Phytochemicals such as polyphenols have been found to stimulate the non-enzymatic antioxidant system by altering the peroxidation kinetics and neutralizing ROS such as free radicals, singlet oxygen, and triplicate oxygen [53]. The present study revealed significant increases in DPPH and FRAP activities in cultures following treatment with all elicitor concentrations. Treatment with 100 mg/L caused the maximum scavenging activity of the free radicals produced (Figure 3). It is likely that the increased antioxidant activity in shoot cultures of *P. tuberosa* in response to elicitors was due to antioxidant phytochemicals and their constructive interactions in the presence of elicitor molecules. There is evidence that antioxidant capability is dependent on the type and location of hydroxyl groups, as well as the type of polyphenol [54]. Polysaccharides have putative effects on binary combinations of polyphenol compounds [55,56], which may contribute to increased antioxidant activity under elicitor treatments. The hydrogen-donating ability of accessible antioxidants has been hypothesized to explain their ability to scavenge free DPPH and ferric radicals [57,58]. In response to different elicitors, TP and TF would be reliable markers for measuring antioxidant activity and secondary metabolites. These observations further support the results of Ullah et al. [59], who showed that polysaccharide elicitors significantly improved antioxidant activity in microshoot cultures of *Ajuga integrifolia* through higher scavenging of free radicals. 

Isoflavones are plant defense molecules; therefore, elicitation could be a viable way to increase their production in vitro. In the present study, shoots treated with 100 mg/L PEC showed enhanced total isoflavonoid content compared to the control shoots and mother plant shoots. An increase in the amount of total isoflavonoid content after elicitation with polysaccharides is often correlated with the up-regulation of key genes related to isoflavonoid biosynthesis [60,61]. Similar evidence of an increase isoflavonoid content was also observed in poly- or oligosaccharides treated in vitro cultures of *Pueraria candollei* [62], *Nasturtium ofcinale* [63], and *Stevia rebaudiana* [64]. 

## 4. Materials and Methods

### 4.1. Plant Materials

In vitro propagated shoot cultures of *P. tuberosa* were used as plant material (data not published, communicated). The 250 mL Borosil glass conical flasks with 80 mL MS medium (control and elicitors treated) and 3 nodal segments of in vitro grown shoots as the inoculum were used as experimental set up.

### 4.2. Preparation of Elicitors

YE, PEC, and ALG (Himedia) were dissolved in double distilled water and the pH of the elicitor solutions was adjusted to 5.8. The stock solution of ALG was filter-sterilized through a 0.45 mm syringe Millipore filter (Axiva, India) and then added to the autoclaved MS media at the desired concentrations under aseptic conditions on a Laminar Air Flow Bench. Stock solutions of YE and PEC were added to the medium at the desired concentrations before autoclaving at 121 °C for 20 min.

### 4.3. Elicitor Treatment and Culture Conditions

To conduct elicitation experiments, in vitro grown shoots (1.0 to 1.5 cm) with one node were used as explants. MS basal medium containing 3% (*w*/*v*) sucrose, 0.8% (*w*/*v*) agar, 0.57 µM/L TDZ, and 0.12 µM/L IBA, with pH adjusted to 5.84, was used for elicitation experiments. For elicitation, YE, PEC, and ALG at concentrations of 0.05–0.2 g/L were added to the media. All cultures were maintained in a growth room at 25 ± 1 °C under cool white fluorescent light with a light/dark photoperiod of 16/8 h. After 4 weeks of treatment, all shoots were harvested.

### 4.4. Study of Growth 

Growth parameters such as the total number of shoots (TNS), fresh weight (FW), and dry weight (DW) in the presence of all three elicitors mentioned above were studied. The number of shoots and their FW and DW were measured on the basis of per liter MS solution.

### 4.5. Estimation of the Chlorophyll, Protein and Carbohydrate Content

Chlorophyll content (Chl a, Chl b and Total Chl) was assessed according to the method described by Arnon [65]. The Bradford method [66] was used to determine protein content where bovine serum albumin was used as the standard. A modification of Anthrone’s method was employed to determine the carbohydrate content [67].

### 4.6. Determination of TP, TF Content and Antioxidant Activity

#### 4.6.1. Sample Preparation

The in vitro elicited shoots were dried at 60 °C for 12 h in an oven and then ground in a pastel motor. The extract was prepared (cold extraction method) by dissolving 250 mg of dried powder of in vitro elicited shoots in 5 mL 70% methanol and shaking on a test tube rotator (Model Abdo’s waves) for 12 h at 70 rpm at room temperature (24–26 °C). Following sonication for 10 min using a sonicator (Sonar), the sample was centrifuged for 10 min at 3000× *g*. The supernatant was collected and stored at 20 °C until further analysis.

#### 4.6.2. Determination of TP Content

The TP content was measured by the method of Farkas and Kiraly [68]. The reaction mixture (total volume 3 mL), consisting of 0.2 mL extract, 0.2 mL of 50% Folin–Ciocalteu reagent, and 0.4 mL of 20% Na_2_CO_3_, was shaken vigorously with various samples. After incubation at 100 °C in a water bath for 1 min, the solution was cooled under running tap water for 15 min at room temperature, and the blue color solution was mixed with 4.2 mL of double distilled water. The TP was expressed as mg gallic acid equivalents (GAE g/DW) using a calibration curve with gallic acid at 650 nm.

#### 4.6.3. Determination of TF Content

The TF content was estimated using an aluminum chloride colorimetric assay [69]. The reaction mixture (total volume 10 mL) consisting of 1 mL sample of prepared extract or standard was mixed with 4 mL distilled water and 0.3 mL of 5% (*w*/*v*) NaNO_2_. After 5 min and 1 min, 0.3 mL of 10% (*w*/*v*) AlCl_3_ and 2 mL of 1 M NaOH were added, respectively. Then, 2.4 mL of distilled water was added, and the mixture was shaken. The resultant pink color was read using a spectrophotometer at 510 nm, and the results were expressed in mg quercetin equivalents (QE) per g DW sample.

#### 4.6.4. DPPH Radical Scavenging Activity

The DPPH radical scavenging activity was determined according to the method described by Hatano et al. [70]. The reaction mixture (total volume 3 mL), consisting of 0.1 mL extract, 0.5 mL of 0.5 M acetic acid buffer solution at pH 5.5, 1 mL of 0.2 mM DPPH in ethanol, and 1.4 mL of 50% (*v*/*v*) ethanol aqueous solution, was shaken vigorously with various samples. After incubation at room temperature for 30 min, the remaining DPPH was determined by measuring the absorbance at 517 nm, and the radical scavenging activity of each sample was expressed as the ratio of the decrease in absorption of DPPH radical scavenging activity (%) to that of the control DPPH solution (100%) in the absence of the sample.

$$\mathrm{DPPH}\;\mathrm{radical}\;\mathrm{scavenging}\;\mathrm{activity}\left(\%\right)=\frac{\left(A-B\right)}{A}\times 100$$

A = absorption of the control (50% ethanol and without DPPH), B = corrected (50% ethanol and with DPPH) absorption of the sample reaction mixture.

#### 4.6.5. SOD Activity

Superoxide anions were generated using a phenazine methosulphate (PMS)/nicotinamide adenine dinucleotide sodium salt (NADH) system. The SOD activity was determined according to the method described by Jain et al. [71]. The reduction of nitroblue tetrazolium (NBT) by superoxide anions yields a chromogenic product, which is measured at 560 nm. A total of 10 milligrams of the extract was dissolved in 0.1 mL methanol and volume was increased up to 10 mL with 0.1 M phosphate buffer (pH 7.4). A volume of 62.5 µL of test sample/positive control (various concentrations) in 0.1 M phosphate buffer pH 7.4, 62.5 µL of 468 µM NADH solution, 62.5 µL of 150 µM NBT solution, and 62.5 μL of 60 µM PMS solution were added to a microwell plate and incubated at room temperature for 5 min. Absorbance was measured at 560 nm, and the percentage inhibition of superoxide anion generation was calculated using the same DPPH activity formula.

#### 4.6.6. FRAP Assay

The ferric reducing power of the plant extracts was determined using the slightly modified method of Benzie and Strain [72]. The reaction mixture, containing 100 µL of sample solutions, 300 µL of deionized water, and 3 mL of FRAP reagent, was incubated for 30 min at 37 °C in a water bath and read at 593 nm. The FRAP value was calculated as the difference between the sample absorbance and blank absorbance. FRAP values were expressed as mM Fe_2_SO_4_/g of the sample.

### 4.7. HPLC Analysis

Sample preparation and HPLC analysis were performed as previously described method by Kanthaliya et al. [1]. 

### 4.8. Statistical Analysis

Each experiment was repeated thrice, with 10 explants in each experiment. A total of 30 explants per treatment were evaluated. Results are reported as the mean ± standard deviation (SD) and analyzed by ANOVA followed by Duncan’s multiple range tests (DMRT) at *p* ≤ 0.05, using Statistical Package SPSS Statistics software (version 17.0). OriginPro 2023 (OriginLab, Northampton, MA, USA) was used to create the PCA to estimate the correlation coefficient between the physio-biochemical parameters. 

## 5. Conclusions

The present study demonstrated that elicitors (YE, PEC, and ALG) applied to in vitro propagated shoot cultures of *P. tuberosa* stimulated cellular metabolism, resulting in increased chlorophyll content, primary metabolites (mainly protein, carbohydrates, and secondary metabolites, including TP and TF), and antioxidant activity when compared to untreated in vitro plantlets. Maximum biomass, TP, and TF content as well as antioxidant activity occurred in in vitro shoots treated with 100 mg/L PEC for 4 weeks. On the other hand, ALG 200 mg/L treated shoots were found to contain the maximum amounts of chlorophyll, protein, and carbohydrates. Overall, the results indicate that elicitors YE 200, PEC 100, and ALG 200 mg/L, in particular, can be effective stimulants for growth, antioxidant activity, and the production of bioactive compounds in *P. tubertosa*. The results of the present study provide a roadmap for further studies aimed at obtaining phytopharmaceutical advantages under various biotic and environmental influences. Currently, nanoscale elicitors are used to stimulate defense pathways and secondary metabolites in in vitro cultures. The current study can be extended using this novel approach to assess the role of targeted elicitors at the nanoscale in the secondary metabolite pathway.

## Figures and Tables

**Table 1:** Effect of elicitor treatments on chlorophyll, protein, and carbohydrate content in *P. tuberosa* shoots. Note: Values are mean ± SD, means in a column followed by different letters are significantly different from each other at *p* < 0.05. Mean values followed by the same letter(s) in a column are not significantly different (*p* < 0.05) based on DMRT.

Concentration	Elicitor	Chlorophyll (mg/g)			Protein (mg/g)	Carbohydrate (mg/g)
		Chl a	Chl b	Total Chl		
0 mg/L	Control	17.4 ± 0.35 ^cde^	11.96 ± 0.47 ^de^	29.36 ± 0.41 ^fg^	8.29 ± 0.78 ^e^	6.55 ± 0.33 ^d^
50 mg/L	YE	17.81 ± 0.56 ^cd^	11.25 ± 0.13 ^e^	28.06 ± 0.74 ^gh^	9.52 ± 0.64 ^de^	9.18 ± 0.14 ^bc^
	PEC	22.58 ± 0.47 ^b^	12.59 ± 0.15 ^de^	30.17 ± 0.19 ^ef^	10.74 ± 0.58 ^cd^	9.83 ± 0.17 ^ab^
	ALG	12.26 ± 0.36 ^f^	13.58 ± 0.19 ^d^	32.45 ± 0.57 ^cd^	10.29 ± 0.37 ^cd^	7.01 ± 0.12 ^d^
100 mg/L	YE	15.82 ± 0.45 ^e^	13.31 ± 0.85 ^d^	27.12 ± 0.52 ^h^	10.71 ± 0.29 ^cd^	10.04 ± 0.57 ^ab^
	PEC	27.55 ± 0.15 ^a^	16.71 ± 0.21 ^bc^	34.26 ± 0.51 ^c^	12.87 ± 0.62 ^b^	11.1 ± 0.18 ^ab^
	ALG	16.16 ± 0.25 ^de^	16.02 ± 0.14 ^bc^	42.17 ± 0.46 ^b^	12.21 ± 0.22 ^bc^	7.63 ± 0.78 ^cd^
200 mg/L	YE	18.84 ± 0.77 ^c^	17.64 ± 0.47 ^ab^	31.16 ± 0.91 ^de^	11.25 ± 0.91 ^bcd^	11.1 ± 0.78 ^ab^
	PEC	26.02 ± 0.35 ^a^	15.46 ± 0.29 ^c^	31.48 ± 0.34 ^de^	9.53 ± 0.42 ^de^	10.22 ± 0.78 ^ab^
	ALG	18.28 ± 0.41 ^c^	18.87 ± 0.09 ^a^	48.15 ± 0.39 ^a^	18.66 ± 0.17 ^a^	11.46 ± 0.78 ^a^

**Table 2:** Loading matrix for principal components.

Traits	PC1	PC2
TNS	0.311	−0.231
FW	0.310	−0.210
DW	0.289	−0.135
Chl a	0.263	−0.352
Chl b	0.256	0.377
Total Chl	0.168	0.458
Protein	0.203	0.494
Carbohydrates	0.254	0.138
TP	0.313	−0.229
TF	0.314	−0.195
DPPH	0.298	0.129
FRAP	0.337	0.032
SOD	−0.231	−0.186

## Data Availability

Not applicable.
